# Identification of a Highly Virulent *Verticillium nonalfalfae* Strain Vn011 and Expression Analysis of Its Orphan Genes During Potato Inoculation

**DOI:** 10.3390/plants14091281

**Published:** 2025-04-23

**Authors:** Mengyuan Wan, Xinlong Chen, Xiaoxi Yi, Yi Fu, Yuanliang Jin, Dianqiu Lyu

**Affiliations:** 1 Integrative Science Center of Germplasm Creation in Western China (CHONGQING) Science City, College of Agronomy and Biotechnology, Southwest University, Chongqing 400715, China; 18306070359@163.com (M.W.); slndchenxinlong@163.com (X.C.); 2 Key Laboratory of Germplasm Innovation of Upper Yangtze River, Ministry of Agriculture and Rural Affairs, Chongqing 400715, China; 3 Chongqing Key Laboratory of Biology and Genetic Breeding for Tuber and Root Crops, Chongqing 400715, China; icecream20067@126.com (X.Y.); 15086689332@163.com (Y.F.);

**Keywords:** potato (*Solanum tuberosum* L.), Verticillium wilt, *Verticillium nonalfalfae*, orphan genes, transcriptome analysis

## Abstract

Potato (*Solanum tuberosum* L.) is an important food crop and in recent years, Verticillium wilt has become one of the major diseases limiting potato production. To study potato Verticillium wilt, a highly pathogenic strain was isolated from field samples in Heilongjiang. After sequencing and morphological identification, the strain was confirmed as a host-specialized *Verticillium nonalfalfae* (*V. nonalfalfae*), Vn011. The genome analysis revealed 151 orphan genes in Vn011, and comparative transcriptomic analysis before and after potato inoculation showed differential expression of 21 genes, including several encoding low-complexity regions (LCRs) and transmembrane (TM) domains. These domains are known to be involved in pathogen signaling, protein folding, and phase separation processes. This study presents the whole-genome sequence of Vn011, having predicted and analyzed the expression changes of orphan genes during the infection process of *V. nonalfalfae* in potato, and provides new insights into the pathogenic mechanisms of the pathogen. Further research on these orphan genes will not only enhance the understanding of the evolutionary adaptation of *V. nonalfalfae*, but may also provide new molecular targets for the control of potato wilt disease.

## 1. Introduction

Potato (*Solanum tuberosum* L.) is an annual herbaceous plant of the *Solanaceae* family, originating from the Andes Mountains in South America. Due to its high yield, rich nutritional value, and strong adaptability, it has become the world’s fourth most important food crop after rice, wheat, and maize [1,2]. The advantages of potato are not only reflected in its high yield and broad environmental adaptability but also in its strong tolerance to poor soil conditions [3,4]. However, with the expansion of the planting area and the increase in cultivation years, the limitation of arable land has led to the frequent occurrence of continuous cropping, further exacerbating the spread of soil-borne diseases. Among these, potato Verticillium wilt has gradually become one of the major constraints to the sustainable development of the potato industry [5].

Potato Verticillium wilt was identified as a key disease affecting the potato industry as early as 1967. In 1987, the pathogenic fungus was first isolated from potato plants in Germany and was confirmed to induce Verticillium wilt [6]. In Canada, the occurrence of Verticillium wilt was first recorded in 1987, and in recent years, it has become a significant obstacle to local potato production [7]. Since the first case of Verticillium wilt was discovered in Sichuan Province, China, the disease has become increasingly severe in seven other provinces (autonomous regions), including Inner Mongolia, Hebei, Gansu, Heilongjiang, Shanxi, Ningxia, and Yunnan, posing a significant threat to local potato production [8].

Verticillium wilt, a characteristic soil-borne and seed-borne vascular disease, is predominantly caused by fungi from the *Verticillium* genus, with ten species identified as key culprits [9,10]. Fungi of the *Verticillium* genus have a broad host range and can infect various plants, including chrysanthemum, sunflower, cotton, rapeseed, cucumber, potato, and *Nicotiana benthamiana*. The primary pathogens responsible for Verticillium wilt symptoms include *V. albo-atrum*, *V. alfalfae*, *V. longisporium*, *V. dahliae*, and *V. nonalfalfae*. Among them, *V. dahliae* and *V. nonalfalfae* are the major pathogens causing potato Verticillium wilt [11,12]. Although *V. nonalfalfae* has been relatively less studied in Verticillium wilt research, an increasing number of studies in recent years have reported its pathogenic effects on various crops, including hops, kiwifruit, and *Ailanthus altissima* [13,14,15].

Due to the widespread transmission of Verticillium wilt pathogens and the still unresolved complexity of their infection mechanisms, the prevention and control of potato Verticillium wilt remain highly challenging. Current research mainly focuses on the regulation of pathogen growth and development, cell wall degradation, effector protein interactions, and virulence [16,17]. However, a substantial number of virulence factors that may participate in or modulate the pathogenic process and potentially play pivotal roles remain insufficiently characterized and largely unexplored. Among them, orphan genes may represent a potential category. The investigation of these unknown factors offers new directions for elucidating the pathogenic mechanisms of Verticillium wilt pathogens.

Orphan genes refer to genes that are present in certain species but lack significant homologs in other species. These genes are often not identified through conventional genome alignment methods and typically lack recognizable evolutionary ancestry, thus are considered as newly emerged, species-specific genes within the genome [18,19,20]. The study of orphan genes is of great significance for uncovering species-specific biological traits, adaptive evolution, and the functional diversity of genes [21,22,23]. In fungi, orphan genes are often closely associated with their ecological adaptability, metabolic characteristics, and interactions with hosts. For example, orphan genes can be used to rapidly identify fungal species [24]. Fungi may evolve unique orphan genes during interactions with plants, animals, and other microorganisms to enhance their survival and reproduction in specific environments. This is particularly evident in pathogenic microbes, where plant-pathogenic fungi may evolve novel orphan genes to enhance virulence and facilitate infection. Moreover, orphan genes shared across pathogens tend to have a stronger correlation with pathogenicity-related genes [25]. In the co-evolutionary arms race between pathogens and their hosts, orphan genes play a crucial role, continuously evolving to enhance pathogenicity or defense mechanisms [26].

We obtained a highly virulent strain of Verticillium causing potato wilt disease from fields in Heilongjiang. After isolation from plants, morphological and genomic identification confirmed that it is *V. nonalfalfae*, and we designated it as Vn011, with potato as its primary host. Through genomic and transcriptomic analyses, we identified 151 orphan genes in Vn011, with 21 differentially expressed genes before and after potato inoculation. These findings provide theoretical support for research on potato Verticillium wilt resistance.

## 2. Result

### 2.1. Isolation of a Highly Virulent Verticillium Wilt Pathogen from Potato Fields

**Figure 1:**
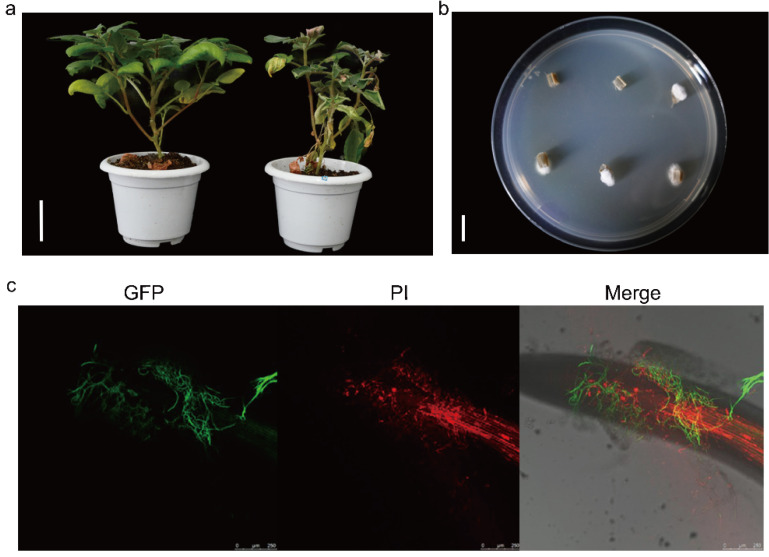
Pathogen invasion in potato. (**a**) Healthy and diseased potato plants. Fungi isolated from field-grown potatoes were inoculated in a laboratory experiment, leading to severe disease in the potatoes. Scale bar = 5 cm. (**b**) Pathogenic fungi isolated from infected potato stems. Pathogens were isolated from diseased potato plants and cultured on PDA medium for one week. Scale bar = 1 cm. (**c**) Confocal microscopy observation of GFP-labeled pathogens infecting potatoes. After co-culturing the fungus on water agar medium for 48 h, the infection process in potatoes was observed.

**Figure 2:**
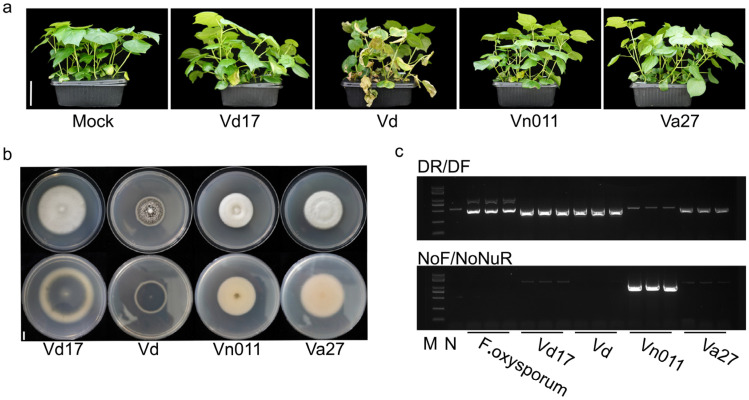
PCR identification of *Verticillium* species and pathogenicity on cotton. (**a**) Inoculated cotton plants with *Verticillium* species. Cotton plants were inoculated with Vd17, Vd, Va27, and Vn011, and pathogenicity was assessed 30 days post-inoculation. Mock is the control treated with sterile culture solution. Scale bar = 10 cm. (**b**) Colony morphology of Verticillium species. Colony morphology of Vd17, Vd, Va27, and Vn011 after 10 days of growth on PDA medium. Scale bar = 1 cm. (**c**) Specific primers for Verticillium species identification. PCR identification of *Fusarium oxysporum*, Vd17, Vd, Va27, and Vn011 using species-specific primers for Vd (DR/DF) and Vn (NoF/NoNuR).

An extremely aggressive strain of the potato Verticillium wilt pathogen was isolated from a severely diseased field in Heilongjiang. Infected potato plants were collected, and pathogen isolation was performed from the basal stem sections of severely affected plants. After culturing on PDA medium at 22 °C in darkness for 7 days, white mycelia emerged from the disinfected plant stem tissues (Figure 1b). Following aseptic single-spore isolation, a pathogenic fungal strain was obtained. The strain produced white, cottony colonies characterized by profuse aerial mycelia and well-defined, regular margins when cultured on PDA medium (Figure 2b). Its morphological characteristics were consistent with the typical description of *V. nonalfalfae* [9]. Following inoculation under laboratory conditions, the strain caused yellowing of potato leaves, followed by progressive wilting of the stem and eventual leaf withering, ultimately leading to plant death (Figure 1a). Therefore, this strain was determined to be highly pathogenic. Subsequently, GFP transformation was used to label the strain for observing fungal infection in potato seedlings. At 48 h post-inoculation, fungal hyphae elongated longitudinally along the intercellular spaces of root epidermal cells, forming a dense hyphal network around the root base. The fungus entered the plant through the roots and successfully colonized the tissues (Figure 1c). No fluorescence signal was detected in the GFP channel when examining uninoculated potato roots without pathogen (Figure S1). Based on morphological characteristics, pathogenicity phenotypes, and infection dynamics, this strain was identified as *V. nonalfalfae*.

### 2.2. Identification of V. nonalfalfae via PCR and Sequencing Analysis

To confirm whether this strain is the causal agent of potato Verticillium wilt, we first performed PCR amplification using *Verticillium*-specific primers. PCR was conducted alongside *Fusarium oxysporum*, *Verticillium dahliae*, and *Verticillium alfalfae* strains preserved in our laboratory (Figure 2b,c). The results demonstrated that Vn011 yielded a distinct DNA band of the expected size when amplified with the *V. nonalfalfae*-specific primers NoF/NoNuR. However, similar bands were also observed in other *Verticillium* species. Additionally, when using *V. dahliae*-specific primers DR/DF, Vn011 did not produce the expected 490 bp band, though non-*V. dahliae* strains showed similar bands. These results suggest that the strain can be preliminarily identified as *V. nonalfalfae* based on these primers.

**Figure 3:**
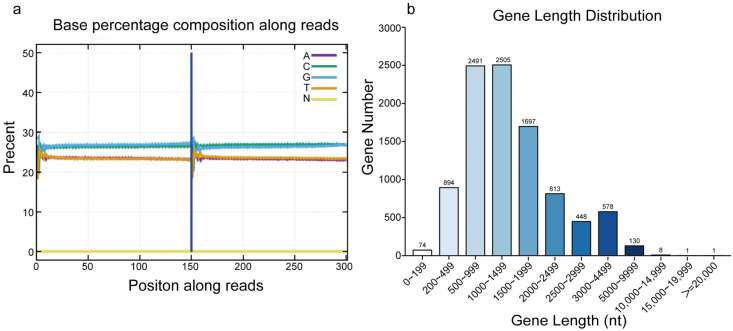
Quality control of resequencing data and gene features. (**a**) Base distribution plot. (**b**) Gene length distribution plot.

Next, whole-genome resequencing of the strain was performed by BGI using its proprietary sequencing platform. The total raw sequencing data amounted to 5258 Mb and passed quality control and base balance assessments (Figure 3a). The genome assembly resulted in a total length of 33,634,619 bp, consisting of 185 scaffolds, with a GC content of 55.08%. A total of 9640 protein-coding genes and 640 non-coding RNAs were predicted, with gene lengths primarily distributed between 500 and 1999 bp, accounting for 69.4% of all genes (Figure 3b).

**Figure 4:**
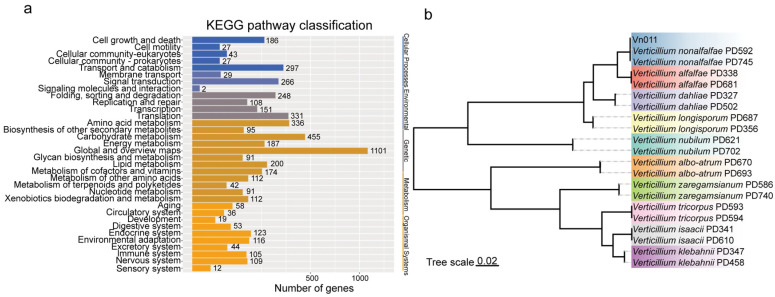
Phylogenetic classification of Vn011. (**a**) KEGG gene annotation. (**b**) Phylogenetic tree of *Verticillium* species. A maximum likelihood tree based on four single-copy conserved genes (*Actin*, *EF-1α*, *GPD*, *TS*) and other *Verticillium* species in the NCBI database.

Among the predicted genes, 3142, 1281, 9551, 4174, and 5728 genes were annotated in the SWISSPROT (Swiss-Prot (UniProt), available online at https://www.expasy.org/resources/uniprotkb-swiss-prot (accessed on 3 April 2025)); COG (Clusters of Orthologous Groups (NCBI), available online at https://www.ncbi.nlm.nih.gov/COG (accessed on 3 April 2025)), NR (Non-redundant Protein Database (NCBI)), available online at https://www.ncbi.nlm.nih.gov/protein (accessed on 3 April 2025)); KEGG (Kyoto Encyclopedia of Genes and Genomes (KEGG), available online at https://www.kegg.jp (accessed on 3 April 2025)); and GO (Gene Ontology (Gene Ontology Consortium), available online at https://geneontology.org (accessed on 3 April 2025)) databases, respectively. KEGG annotations revealed a large number of genes related to pathogenicity and various metabolic pathways (Figure 4a), indicating that this strain possesses essential genes associated with its pathogenic nature. Finally, a maximum likelihood phylogenetic tree was constructed using four single-copy orthologous genes *Actin*, *Elongation Factor 1-alpha (EF-1α)*, *Glyceraldehyde-3-phosphate Dehydrogenase (GPD)*, *Tryptophan Synthase* (*TS*) and homologous genes from other *Verticillium* strains in the NCBI database (Figure 4b). The results showed that Vn011 formed a sister clade with *V. nonalfalfae* strains PD592 and PD745 (Figure 4b).

Based on morphological features, molecular identification, and genomic analysis, this strain was ultimately designated as Vn011 and identified as *V. nonalfalfae*.

### 2.3. Identification of Orphan Genes

**Figure 5:**
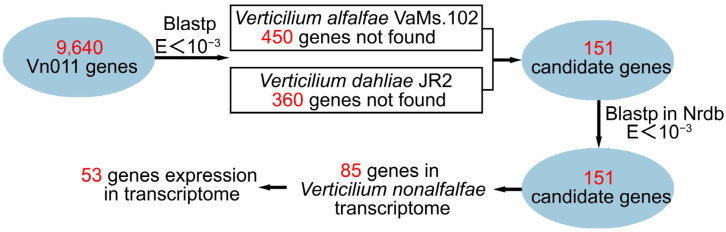
Workflow for screening orphan genes in the Vn011 genome.

The Vn011 strain exhibits strong pathogenicity in potatoes but does not cause disease in cotton, indicating a degree of host specificity (Figure 1a and Figure 2a). This study focuses on orphan genes in Vn011 to determine their potential role in pathogenicity. To identify orphan genes, we screened the Vn011 genome by integrating transcriptomic data and comparative genomics. Using the BLASTP function on the Galaxy platform, we compared 9640 predicted Vn011 genes against the genomes of two other *Verticillium* species with an *E*-value threshold of <10^−3^. The 151 candidate genes are the intersection of genes uniquely present in *V. nonalfalfae* Vn011 but absent in both *V. alfalfae* VaMs.102 (450 unique genes) and *V. dahliae* JR2 (360 unique genes). Among these, 151 genes appeared in both comparisons and had no homologs in the Nrdb database, designating them as candidate orphan genes of Vn011. Further analysis comparing these 151 genes with the *V. nonalfalfae* genome in the NCBI database and transcriptome data revealed that 85 genes had homologs in the *V. nonalfalfae* genome. Only 53 genes were transcriptionally active in the fungal transcriptome before and after potato infection. These findings suggest that these 53 orphan genes may be involved in Vn011 pathogenicity (Figure 5).

### 2.4. Expression Patterns of Orphan Genes After Inoculation

**Figure 6:**
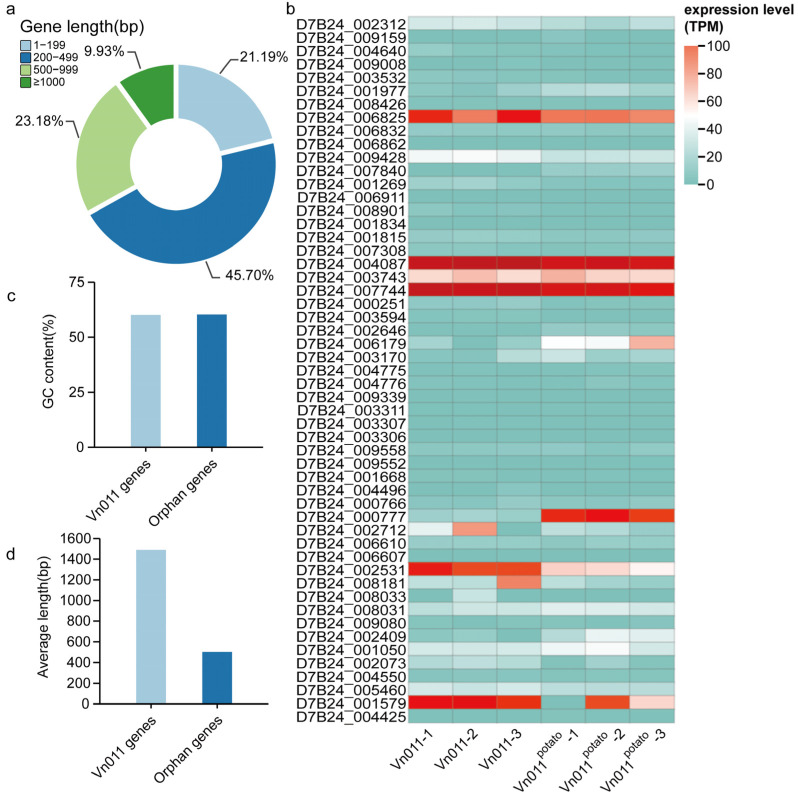
Characteristics and expression profiles of orphan genes in the Vn011 genome. (**a**) Length distribution of orphan genes in Vn011. (**b**) Expression levels of orphan genes in Vn011 at least at one time point (either before or after potato inoculation). (**c**) GC content comparison between orphan genes and all genes in Vn011. (**d**) Average gene length comparison between orphan genes and all genes in Vn011.

A comparison of the 151 orphan genes with all genes from the resequencing data (Figure 6c,d) revealed that orphan genes were generally shorter. Specifically, 45.70% of orphan genes fell within the 200–499 bp range (Figure 6a). The average length of orphan genes (502 bp) was significantly shorter than the average length of all Vn011 genes (1490 bp) (Figure 6d). Notably, GC content showed no significant difference between orphan genes and the full Vn011 genome, averaging 60.27% and 60.08%, respectively (Figure 6c).

**Figure 7:**
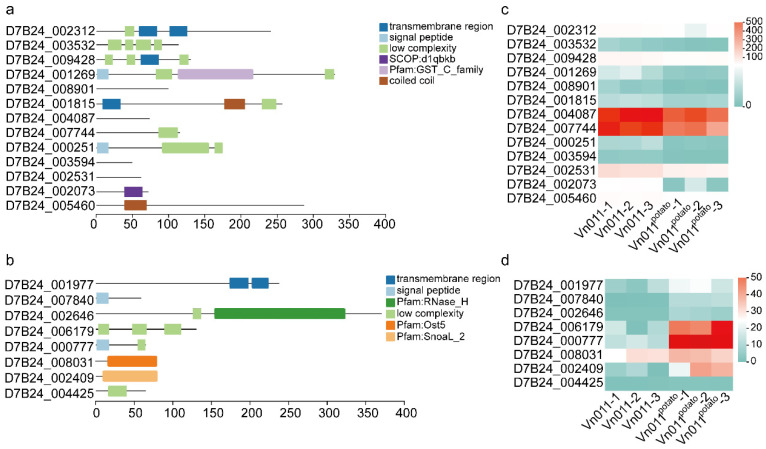
Differentially expressed genes before and after inoculation and domain prediction. (**a**) Domain prediction and distribution for downregulated genes. (**b**) Domain prediction and distribution for upregulated genes. (**c**) Expression level differences in downregulated genes. (**d**) Expression level differences in upregulated genes.

Gene expression patterns often provide insights into gene function. Among the 85 *V. nonalfalfae* orphan genes, a total of 53 genes were expressed at least at one time point (either before or after Vn011 inoculation of potatoes). Most exhibited low expression levels, with only 15 genes showing expression levels exceeding 20 in one or both time points (Figure 6b). Further analysis of the 53 expressed genes identified that 8 genes exhibited upregulation, while 13 genes demonstrated downregulation following inoculation (Figure 7c,d). These expression shifts suggest that a subset of orphan genes might play active roles during Vn011 infection of potatoes, potentially contributing to host-specific pathogenicity.

### 2.5. Functional Features of Orphan Genes

To gain insights into the potential functions of the 21 orphan genes differentially expressed before and after potato inoculation, domain prediction was performed. All 21 genes were classified as “uncharacterized proteins”. A significant portion of these genes contained regions of low complexity. Specifically, 11 of these genes included low-complexity regions (LCRs), and 5 genes (*D7B24_003532*, *D7B24_009428*, *D7B24_001269*, *D7B24_000251*, and *D7B24_006179*) contained two or more of these regions. Additionally, four genes were predicted to have transmembrane (TM) regions, and another four genes were predicted to have signal peptides. Interestingly, four genes did not have any identifiable structural domains, while *D7B24_007840* was predicted to function as an effector protein.

These findings underscore the diversity of orphan genes, as well as the challenges in studying their functions (Figure 7a,b). This variability in gene characteristics further highlights the complexity of identifying and understanding the roles of orphan genes in the host-specific pathogenicity of Vn011.

## 3. Discussion

The *Verticillium* species comprise a group of filamentous fungi that are ubiquitously found in soil. Many of these fungi are pathogenic and represent substantial threats to agricultural productivity. The resequencing of *V. nonalfalfae* genomes enhances the genetic diversity of *Verticillium* species and provides valuable data for evolutionary studies of this genus. Obtaining the genomic information of pathogenic fungi is crucial to understanding their pathogenic mechanisms, immune evasion strategies, drug resistance, and evolutionary processes [27,28,29]. The acquisition of fungal pathogen genome sequences facilitates the identification of pathogenicity factors, including effectors, toxin biosynthetic genes, and other functionally critical genes. Previous studies have demonstrated that key pathogenicity factors can be predicted to elucidate the mechanisms of pathogenesis in *Magnaporthe oryzae* and *V. dahliae* [30,31,32]. In addition to pathogenicity studies, fungal genome sequencing is also essential for investigating antifungal drug resistance [33]. Many fungal pathogens, such as *Candida albicans* and *Botrytis cinerea*, have developed resistance to conventional antifungal treatments [34,35]. In-depth research on antifungal resistance genes enables the identification of novel drug targets, thereby providing theoretical support for the development of new antifungal agents. Furthermore, comparative genomics allows researchers to analyze the phylogenetic relationships among different fungal pathogens, infer pathogenic determinants, and identify novel candidate genes potentially involved in disease progression [36,37,38]. By integrating genomic data with large-scale data analysis, it is possible to monitor changes in pathogen population structures, predict emerging disease outbreaks, and develop effective disease management strategies [39,40]. Through the sequencing of Vn011, we can rapidly predict and identify its associated pathogenic genes, providing a theoretical foundation for the prevention and control of potato Verticillium wilt disease.

Host specialization refers to the ability of pathogenic microorganisms to exhibit high adaptability to specific host populations or organs. This process involves complex interactions between fungi and their hosts, including immune evasion, nutrient acquisition, and the expression of virulence factors [41,42]. The host specificity of fungi not only reflects their adaptation to different host environments but also underscores the fine-scale evolutionary adjustments they undergo [43]. Host specialization is driven by co-evolution between pathogens and their host plants. Rapidly evolving pathogen populations often circumvent plant immune defenses through genetic mutations, recombination, or horizontal gene transfer [44,45,46,47]. The host specificity of Vn011 toward potatoes suggests that the interaction between *V. nonalfalfae* and potatoes should be a key focus of future research. Understanding the genetic exchanges between potatoes and pathogens in the context of pathogen-plant co-evolution will provide deeper insights into host-pathogen interactions.

In pathogenic microorganisms, orphan genes may play crucial roles in species-specific metabolic pathways, adaptive evolution, and host interactions [26,48]. The study of orphan genes provides valuable insights into gene flow between species, speciation processes, and gene selection mechanisms. Unlike conserved genes, orphan genes often exhibit highly specialized functions, contributing to ecological adaptation [49]. They may encode unique enzymes or surface receptors [50], which could facilitate fungal adaptation to host environments or enable immune evasion. The evolutionary mechanisms of orphan genes are highly complex. Many orphan genes arise through genetic mutations, gene amplifications, or horizontal gene transfer [51,52]. The evolution of these genes is often closely associated with environmental pressures, particularly in host–pathogen interactions [53]. Research on orphan genes not only enhances our understanding of how species adapt to different environments but also offers novel perspectives for developing potential drug targets. Orphan gene screening and expression analysis in the Vn011 genome revealed a substantial number of non-expressed orphan genes. These genes may play critical roles under specific stress conditions during pathogen invasion, or they could represent proto-pathogenic genes still undergoing evolutionary refinement.

Among the 21 orphan genes that exhibited significant expression changes before and after Vn011 inoculation in potatoes, 11 contained LCR domains, and 4 harbored TM domains. TM domains are typically found in transmembrane proteins, which play essential roles not only in the basic metabolism of pathogenic organisms but also in host infection processes [54,55]. In plant pathogens, TM-containing proteins often act as effectors or receptors that recognize host cell surface molecules. Notably, the orphan gene *D7B24_001977*, which contains the TM domain, was upregulated after inoculation of potato, which may suggest that it is able to participate in related activities. LCR domains are widely distributed in various proteins and are known to participate in signal transduction, protein folding, and liquid–liquid phase separation [56,57]. LCRs were observed in both induced and repressed genes, suggesting their dual roles in promoting pathogen fitness and modulating host interactions. The frequent occurrence of LCR domains in orphan genes suggests their potential functional significance in gene evolution. While these structural features provide valuable clues about the roles of orphan genes in pathogenicity, further molecular biology experiments are required to validate their functional implications in fungal virulence and adaptation.

## 4. Methods

### 4.1. Plants Cultivation

Aseptic potatoes were inoculated onto MS solid medium. The cultures were maintained in a growth chamber under a photoperiod of 16 h light/8 h dark, with a light intensity of 20,000 Lx and a temperature of 22 °C, for 14 days. Uniformly grown potato plantlets were selected, and the MS medium was removed by washing before acclimatization. Vermiculite and nutrient soil were mixed in a 1:3 ratio and placed into plastic pots. The acclimated potato plantlets were then transplanted into these pots and cultured in an illuminated plant incubator under the same conditions of 16 h light/8 h dark, with a light intensity of 20,000 Lx and a temperature of 22 °C.

### 4.2. Isolation and Purification of Verticillium from Potato

Potato stems were cut into small segments of approximately 0.5 to 1 cm in length. The segments were first rinsed with sterile water to remove surface dust and impurities, then disinfected by soaking in 75% ethanol for 5 min, followed by immersion in 3% sodium hypochlorite solution for 15 min. The disinfected potato stem segments were then rinsed three times with sterile water until all traces of the 84 disinfectant solution was removed. The dried stem segments were placed on sterilized filter paper and subsequently transferred onto PDA medium, where they were incubated upright in a constant-temperature incubator at 22 °C. Once white mycelia appeared on the PDA medium, a portion of the mycelia was picked using a sterile toothpick and suspended in a sterile 2 mL centrifuge tube containing sterile water, followed by vortex mixing. The suspension was then subjected to serial dilutions to 10^−1^, 10^−3^, 10^−5^, and 10^−7^. The diluted spore suspension was spread onto PDA medium using a spreader. After a week, individual colonies that appeared on the PDA plates were selected and transferred to fresh medium for subculture. Morphological characteristics of the isolates were then observed [58,59].

### 4.3. Pathogen Inoculation and Identification

The pathogen isolated from infected potato in the field was cultured and proliferated. DNA was extracted using the CTAB method, and PCR verification was performed using multiple primer pairs [10]. Conserved genes were screened within the genome, and a phylogenetic tree was constructed alongside fungi of the Verticillium genus. For pathogenicity identification, *V. nonalfalfae* was cultured in a constant-temperature incubator at 22 °C for two weeks. The culture was then transferred to Erlenmeyer flasks containing 700 mL of liquid Czapek-Dox medium and incubated on a shaker at 22 °C, 200 rpm, for 4 to 5 days. The resulting spore suspension was collected by centrifugation at 12,000 rpm for 10 min, and the supernatant was discarded to collect the fungal cells. The fungal cells were resuspended in sterile water, and the spore concentration was adjusted to 1.0 × 10^7^ cfu/mL.

For pathogen inoculation, 12 potatoes with uniform growth in a plant growth chamber were selected. The inoculation was performed using a root-drenching method, in which 50 mL of the spore suspension was applied to each potato plant. The control group was treated with an equal volume of sterile water. Between 30 and 60 days post-inoculation (dpi), the pathogenicity of the fungus was assessed based on disease symptoms observed in the potato plants.

### 4.4. Genome Sequencing of V. nonalfalfae

Enriched fungal biomass was sent to BGI for fungus genome sequencing. DNA was extracted from the fungal samples and size-selected fractionated by electrophoresis. Adapters were added for cluster preparation, followed by sequencing using the BGISEQ platform. The raw sequencing data underwent processing, including quality control and data filtering, to obtain clean data. Genome assembly was performed using genome assembly software. Further analyses included genome component analysis and functional gene annotation [60,61,62]. The sequencing data have been submitted to the Genome Sequence Archive (Genome Sequence Archive v3.0 (BGI), available online at http://gsa.big.ac.cn (accessed on 3 April 2025)) under the accession number CRA023586 [63]. The gene annotation of Vn011 was completed by BGI. BGI’s annotation pipeline utilizes Diamond software tailored to the specific characteristics of each database. The protein databases used for annotation include Gene Ontology (GO) releases 2019-07-01; Kyoto Encyclopedia of Genes and Genomes (KEGG) version 89.1; Cluster of Orthologous Groups of proteins (COG) version 2014-11-10; Swiss-Prot release 2019-07, NR (non-redundant protein sequences) version 2019-07-27.

### 4.5. Identification of Orphan Genes in Vn011

The proteomic sequences of *Verticillium* species were retrieved from the fungal genome database (Ensembl Fungi, release 109 (EBI & Wellcome Sanger Institute), available online at https://fungi.ensembl.org/ (accessed on 3 April 2025)). Protein sequences of *V*. *alfalfae* VaMs.102 and *Verticillium dahliae* JR2 were downloaded and analyzed alongside the proteomic data obtained from the resequencing of *V*. *nonalfalfae*. The data were uploaded to the Galaxy (Galaxy Project, available online at https://galaxyproject.org (accessed on 3 April 2025)) platform for analysis. Homology searches were conducted based on previously established methods [64,65], using an *E*-value threshold of 1 × 10^−3^. BLASTP was employed as the sequence alignment tool. Additionally, the sequences were compared against the non-redundant protein database (Non-redundant Protein Database (NCBI), available online at https://www.ncbi.nlm.nih.gov/protein (accessed on 3 April 2025)) to identify genes exclusively present in the *V. nonalfalfae* genome.

### 4.6. Differential Gene Expression Analysis

RNA-Seq data of *V. nonalfalfae* before and after potato inoculation were used for differential gene expression analysis. Three biological replicates were used for RNA sequencing. The raw sequencing data are available under accession number CRA023478. Transcript expression levels were quantified as transcripts per million (TPM) for visualization of normalized expression patterns. Differential expression analysis was conducted using DESeq2 [66]. The RNA-Seq data analysis followed previously established methods [67]. Heatmaps were generated online using Chiplot (Chiplot v2.1, available online at https://www.chiplot.online/ (accessed on 3 April 2025)) [68].

### 4.7. Observation of Pathogen Infection

A GFP-labeled *V. nonalfalfae* transformant was used to inoculate 2-week-old potato tissue culture plantlets of the Désirée variety. The inoculated plants were grown on water agar medium. After 48 h post-inoculation, potato root samples were collected and stained with propidium iodide for 5 min. The localization and infection patterns of the GFP-labeled transformant within the root system were then observed using laser confocal microscopy

## 5. Conclusions

In this study, we have sequenced and analyzed the genome of *V. nonalfalfae* strain Vn011, a highly pathogenic isolate causing Verticillium wilt in potatoes. The identification of 151 orphan genes in the Vn011 genome provides valuable insights into the molecular mechanisms underlying the pathogen’s virulence. Transcriptomic analysis before and after inoculation of potatoes revealed differential expression of several genes, including those encoding LCRs and TM domains, which are known to be involved in pathogen signaling, protein folding, and phase separation. These findings suggest that these structural domains may play critical roles in the infection process of *V. nonalfalfae*. The comprehensive genomic and transcriptomic data from this study offer new perspectives on the pathogenicity of *V. nonalfalfae* and highlight the importance of orphan genes in the evolutionary adaptation of this pathogen. Further investigation into these orphan genes will not only deepen our understanding of the pathogenic mechanisms but also provide potential new molecular targets for the development of effective control strategies for potato wilt disease.

## Data Availability

Data supporting the findings of this study are available within the paper and within GSA database.

## Supplementary Materials

The following supporting information can be downloaded at: https://www.mdpi.com/article/10.3390/plants14091281/s1, Figure S1: Confocal microscopy observation of potatoes without Vn011-GFP.
