# Supplementary figures and images for: Identification of a Highly Virulent Verticillium nonalfalfae Strain Vn011 and Expression Analysis of Its Orphan Genes During Potato Inoculation
Source: Plants (Basel). 2025 Apr 23;14(9):1281. doi: 10.3390/plants14091281 (PMC12073766; doi:10.3390/plants14091281)

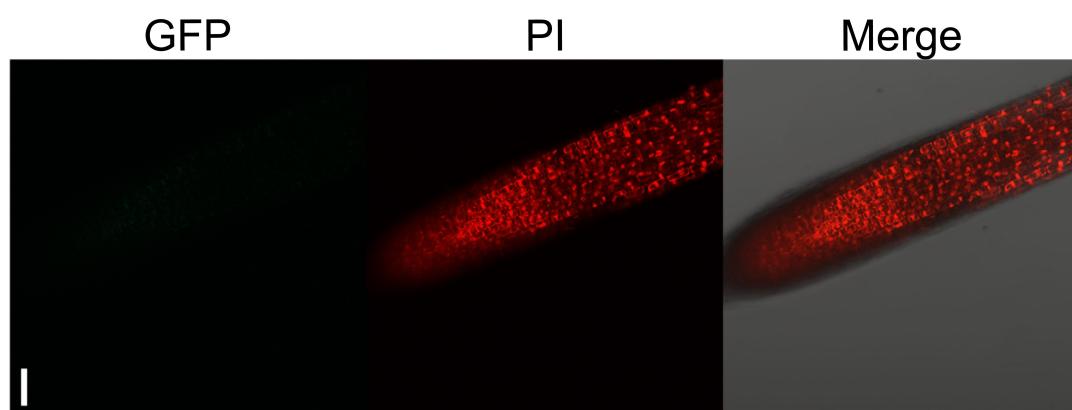

**Figure S1. Confocal microscopy observation of potatoes without Vn011-GFP.**

Supplement: Supplementary file 1 [file plants-14-01281-s001.zip › plants-3565710-supplementary.pdf]
